# Supplementary material for: Are carbon emissions trading and green financial instruments synergistic? -Comprehensive quantitative research based on content analysis
Source: PLoS One. 2024 Mar 7;19(3):e0298601. doi: 10.1371/journal.pone.0298601 (PMC10919601; doi:10.1371/journal.pone.0298601)
Supplement: S1 Data — (ZIP) [file pone.0298601.s001.zip › Data/地级市Z_F工作报告.zip!/─┌├╔╣┼/░n═╖╩╨/└┤╫╘╣1⁄2╓┌║┼í╛┬φ┐╦╩2╛▌═°í┐_2011.docx]

各位代表：

现在，我代表市人民政府向大会报告工作，请连同《包头市国民经济和社会发展第十二个五年规划纲要（草案）》一并审议，并请市政协委员和列席会议的同志们提出意见。

一、“十一五”时期和2010年的工作回顾

“十一五”时期，在自治区党委、政府和市委的正确领导下，我们团结依靠全市各族人民，深入贯彻落实科学发展观，紧紧抓住国家实施西部大开发、自治区促进优势地区率先发展的机遇，坚定不移地推动富民强市进程，有效应对国际金融危机冲击，圆满完成了“十一五”规划确定的主要目标任务，谱写了全市改革开放和现代化建设的新篇章。

——综合经济实力跃升新台阶。五年来，我们坚持发展第一要务不动摇，努力推进经济平稳较快增长，主要经济指标实现重大突破。全市地区生产总值从2005年的861亿元增加到2010年的2460.8亿元，年均增长18.3%，人均地区生产总值近14000美元。规模以上工业增加值由333.6亿元增加到952.4亿元，年均增长25.2%。社会消费品零售总额由292.6亿元增加到720.7亿元，年均增长19.8%。地方财政总收入由99.4亿元增加到243.3亿元，年均增长19.7%。坚持把项目建设作为推动发展的重要载体，五年实施投资5000万元以上重点项目1682个，带动固定资产投资累计完成5913.8亿元，是“十五”时期的4.2倍，建成了一批三次产业、基础设施、环境保护和生态建设、民生以及社会事业的重大项目。

———产业结构调整取得新进展。三次产业结构由2005年的4:52.3:43.7调整为2010年的2.7:54.1:43.2。新型工业化全面推进。钢铁、铝业、装备制造、电力、稀土等优势特色产业得到巩固和提高，实现增加值由198亿元增加到681.7亿元，年均增长28%。其中装备制造业占全市规模以上工业增加值的比重由10%上升到24.5%。风电、光伏产业、新材料等战略性新兴产业迅速成长。风电在建和建成规模达到240万千瓦，并网74万千瓦。已具备年产风力发电主机1900台套和较强的零部件本地配套能力。集约化发展进一步提速。全市工业园区五年累计完成基础设施投资115亿元，收储可利用土地8万亩，入园企业达到1122户，完成工业总产值占全市规模以上工业产值的60%。稀土高新区被国家科技部命名为“国家稀土新材料成果转化及产业化基地”，被国家工信部命名为全国首批“国家新型工业化产业示范基地有色金属（稀土新材料）基地”。全市高新技术产业发展势头良好。世界最大的3.6万吨黑色金属垂直挤压机研制成功，打破了发达国家的技术垄断。具有国际先进水平的包钢百米高速重轨产量占到全国的30%以上，国内直径最大、距离最远的白云鄂博至包钢水及矿浆管道输送工程投入使用，铝钛硼中间合金、生物制柴油等技术完成产业化并投入生产。全市高新技术企业达到58家，占自治区的47%。服务业步入增量提质快速发展阶段。香格里拉酒店、沃尔玛、万达广场相继入驻包头，小肥羊、小尾羊已成为国内外知名品牌，提升了商贸、餐饮等传统服务业发展水平。现代物流业快速崛起，包头跻身全国流通领域现代物流示范城市行列，成为全国17个区域性物流节点城市之一。成功引进浦东发展银行、招商银行、中信银行、华夏银行和内蒙古银行，包商银行发展成为全国性股份制银行。旅游业发展加快，2010年旅游总收入达到109.8亿元，是“十五”末期的4倍。成功举办了中国市长论坛、中国包头·稀土产业论坛、中国制造业论坛等大型活动，进一步提高了城市的知名度和影响力。农牧业综合生产能力稳步提高。菜、薯、乳、肉四大主导产业持续快速发展，农业综合机械化水平提高到72%。蔬菜保护地和设施马铃薯面积分别增加到5.5万亩和53万亩。奶牛和羊规模化饲养水平分别达到70%和50%，良改率分别达到100%和40%。全市规模以上农畜产品加工、流通企业达到160家，实现销售收入227亿元，比“十五”末期增长60%。

——环境保护和生态建设有了新成效。节能减排扎实推进。提前一年完成“十一五”工业节能目标，预计万元工业增加值能耗五年累计下降37%。加大污染物减排力度，如期完成自治区下达的二氧化硫和化学需氧量减排任务。黄河湿地保护、大青山南坡绿化、山北草原围封禁牧三大生态工程实施成效明显。对境内220公里黄河沿线湿地全面保护，推动大青山生态应急水源工程、东河和昆河一期综合整治工程建设，黄河路坝一体工程竣工投入使用。加快实施黄河流域污染治理项目，黄河三断面水质达标率均达到100%。加强大青山国家级自然保护区建设，开展重点区域及大青山沿线砂料场、城区周边地区煤场的综合治理。五年完成造林重点工程211.1万亩，对城郊结合部和大青山南坡10万亩宜林地进行绿化，全市森林覆盖率由9.25%提高到15.1%。实施围封禁牧战略，加强对山北地区2400万亩草场保护，转移安置6620户、2万农牧民，草场植被盖度达到28%，较禁牧前提高5.7个百分点，草原生态环境逐步好转。全市环境质量有效改善，2010年，空气质量二级以上良好天数达到316天，比“十五”末期增加60天。

——统筹城乡发展迈出新步伐。编制完成《城市总体规划（2008—2020）》以及一批重点区域和园区的控制性详规，全市规划建设用地面积达到322平方公里，控制性详规覆盖面积达到257平方公里。五年实施城建项目750个，完成投资超过1000亿元。城区道路、供水排水管网、集中供热等基础设施工程相继竣工投入使用。全市人均道路面积达到12.5平方米，城市日供水能力提高到51万立方米。燃气普及率、集中供热普及率、生活污水处理率、再生水利用率分别达到95%、90%、82%和30%，均比“十五”末期提高20个百分点以上。对163个旧小区和64处“夹心房”实施改造，完成400万平方米既有居住建筑节能改造。建成雪鹿啤酒园、包头鹿园、包头乐园、稀土公园等特色主题公园，实施了一批城市园林绿化工程，全市建成区绿化覆盖率达到40%。全面推进新都市中心区、滨河新区、昆区南部区、青山北部区和石拐南部区建设，城市辐射带动能力不断增强。加强交通网络建设，累计投资112.4亿元，新建改建公路3889公里。先后完成210国道、110国道城区段等道路新建改造工程，实施包固一级公路等市区到外五旗县区高等级公路连通工程。一批农村牧区公路项目相继建成，全市行政村通公路率达到100%。包头至西安铁路和大同至包头、包头至惠农铁路电气化改造全线开通。包头机场实施扩建工程并开展迁建前期工作。城乡一体化发展规划开始实施。“城中村”、城乡结合部改造进一步加快，地区生产生活环境得到改善。切实加大支农支牧力度，五年累计投入财政资金54亿元，是“十五”时期的4.9倍。200个新农村新牧区试点村累计投入资金10.5亿元，95%以上的试点村实现了安全饮水、通油路、通有线电视、有卫生室等目标，试点村农牧民人均收入高于当地收入水平。扶贫开发工作全面加强，五年帮助8.34万农村牧区贫困人口脱贫。集中社会力量重点帮扶固阳县工作成效显著，累计直接投入资金物资1.64亿元，引进各类项目资金14.86亿元，实施帮扶项目369项。

——改革开放步入新阶段。国企改革取得丰硕成果。五年完成企业改革重组75户，引进增量资金262亿元，盘活存量资产209亿元，妥善安置职工8万多人。剥离企业办社会、乡镇苏木机构改革、水利工程管理体制改革等一批重大改革攻坚任务圆满完成。优化资源配置，对全市行政区划进行重大调整。农村牧区综合改革全面推行，集体林权制度改革进入确权发证阶段。事业单位分类改革和人事分配制度改革积极推进，国库集中支付、部门预算全面实行，文化体制改革进一步加快，医药卫生体制改革启动实施。外向型经济加快发展。五年引进国内（区外）资金1520亿元，是“十五”时期的3.4倍，实施各类项目565项。累计完成进出口总额86.2亿美元，其中出口54.5亿美元，是“十五”时期的3.2倍。民营企业健康发展，2010年非公有制经济对全市经济的贡献率达到50.5%。

——人民群众生活得到新改善。财政用于改善民生方面的投入持续增加，五年全市民生支出达到345.1亿元，占地方财政总支出的45%。2010年城镇居民人均可支配收入和农牧民人均纯收入分别达到25862元和8766元，比“十五”末期增长95.7%和87.8%。累计实施好事实事项目78件162项，切实解决了一批人民群众关注的热点难点问题。加大就业再就业工作力度，五年新增城镇就业26.5万人。社会保障体系日臻完善。基本养老和医疗保险实现了对城乡居民政策性全覆盖，城镇基本医疗保险参保率和农牧民养老保险参保率分别达到95%和55.1%，新型农村牧区合作医疗参合率达到98%。全市企业退休人员养老金月人均达到1506元。累计救助城市和农村牧区低保对象409万人次和23万人次，支出保障金7.3亿元和2亿元。救助受灾群众35.3万人次，发放救灾资金6898.3万元。加大保障性住房建设力度，开工建设经济适用住房870万平方米、廉租住房47.7万平方米，建设棚户区安置房108万平方米，改造农村牧区危房4103户。石拐棚户区改造和包钢尾矿坝周边五村搬迁工程全面完成，1.6万户、4万多居民正陆续迁入新居。

——社会事业发展呈现新局面。加快科技事业发展，五年累计投入研发经费28亿元，实施68项重大科技项目，获得国家及省部级以上科技奖250项，我市成为首批国家20个创新型试点城市之一。认真落实“两免一补”政策，五年投入教育经费97.2亿元，是“十五”时期的4.1倍。全面推进中小学校舍安全工程和标准化学校建设工程，实施职业教育基地建设，促进了各类教育均衡发展。新建改建近200所基层医疗卫生机构，建设了一批大型综合医院和专科医院新大楼，城乡医疗卫生服务网络不断完善。加强基层文化基础设施建设，建成34个乡镇综合文化站和422个“草原书屋”。科技少年宫投入使用，新图书馆和少年宫已经落成，“走西口”文化大型城市雕塑组群和传媒大厦等一批公共文化设施加紧实施。送节目下基层活动、农牧民电影放映工程深入推进。广播电视混合覆盖率达到98.5%以上，高于全国全区平均水平。群众体育和竞技体育加快发展，我市代表团在自治区第十二届全运会等重大赛事中取得优异成绩。体育馆建成投入使用，体育场即将竣工。不断加强人事人才工作，人才队伍结构持续优化。人口和计划生育工作连续多年获得自治区表彰奖励，人口自然增长率稳定控制在5％。以内。全面加强精神文明建设，巩固和发展了全国文明城市创建成果。认真落实党的民族宗教政策，促进了各民族团结进步、共同繁荣发展，我市荣获全国民族团结进步模范集体荣誉称号。审计、统计、人防、地震、档案、气象、外事侨务、老龄、史志、仲裁、残疾人事业、红十字会等各项工作都取得了新的成绩。

——政府自身建设再上新水平。认真落实依法治市方略，不断提高依法行政能力。自觉接受人大法律监督和政协民主监督，认真听取各民主党派、工商联和无党派人士意见，促进了决策的科学化和民主化。政府机构改革圆满完成，公务员队伍建设不断加强。切实抓好行政监察和审计监督，重视源头治理，勤政廉政建设取得新成效。政府职能加快转变，社会管理和公共服务能力不断提升。健全完善突发公共事件应急体制机制，妥善处置了禽流感、甲型H1N1流感等突发公共事件。强化信访工作，集中解决了一批群众反映强烈的突出问题。依法严厉打击各类刑事犯罪，维护了社会稳定。坚持标本兼治，安全生产水平稳步提高。加强市场监管和专项整治，确保产品质量和食品药品安全。坚持军政军民团结，双拥工作走在全国全区前列。

刚刚过去的2010年，包头更是在挑战中奋力前行。面对复杂多变的宏观形势，我们把工作的着力点放在转方式、提质量、惠民生、促和谐上，推动经济社会全面持续健康发展。全市地区生产总值、固定资产投资、工业增加值、地方财政总收入等主要经济指标均保持两位数增长。全年实施投资5000万元以上重点项目718个，完成投资1258.3亿元。世界首套、全球最大的神华包头煤制烯烃项目建成并试车成功。全市第三产业增加值首次突破千亿元大关，达到1062.9亿元。高度重视“三农三牧”工作，认真落实国家、自治区各项支农惠牧政策，农牧业生产稳步推进。加快城市建设步伐，一批基础设施项目陆续建成，包头至树林召高速公路黄河特大桥竣工并投入使用，集包铁路增建第二双线工程有序推进，城市轨道交通、呼包高速公路八车道扩建等项目前期工作全面展开。城市公交“一卡通”开通运行。创建国家环保模范城市、国家卫生城市和全国文明城市标兵工作积极推进。举办世界中学生排球锦标赛取得圆满成功。包头第二次荣获“全国城市信息化50强”，并进入前10名行列。以改善民生为重点的社会建设取得新成效，为人民群众兴办的20件38项好事实事全部完成。经过一年的不懈努力，我们为“十一五”发展划上了圆满的句号。

经过五年的实践，我们更加深刻地体会到，实现经济社会又好又快发展，必须始终立足包头实际，更加自觉地置身于全国全区发展的大局中，不断创新发展理念，完善发展思路，努力走出一条老工业基地城市科学发展的现实路径；必须坚持项目带动战略，保持适度投资规模，推进重点产业、基础设施、生态环境、社会事业和改善民生项目建设，提高发展的协调性和可持续能力；必须把提升城市整体形象和品位作为增强综合竞争力的基础和保障，统筹城乡区域协调发展，提高城镇化水平，加强环境保护和生态建设，改善人居环境和发展环境，不断增强包头的辐射带动能力；必须坚持以人为本，努力解决好人民群众最关心、最直接、最现实的利益问题，把发展成果更多更好地惠及人民群众，凝聚全面建设小康社会的强大合力；必须倍加珍惜民族团结、社会和谐的大好局面，始终坚持各民族共同团结奋斗、共同繁荣发展，为改革开放和现代化建设创造和谐稳定的社会环境。

各位代表！回顾“十一五”时期的发展，我们为包头发生的巨大变化而骄傲，为全市各族人民创造的辉煌业绩而自豪！这是自治区党委、政府和市委正确领导的结果，是市人大、市政协和社会各界监督支持的结果，是全市各族人民团结奋斗的结果。在此，我代表市人民政府，向辛勤工作在各条战线上的全市各族人民，向人大代表、政协委员，向各民主党派、人民团体，向离退休老领导、老同志，向驻包部队、武警官兵和公安干警，向所有关心支持包头发展的朋友们，表示衷心的感谢和崇高的敬意！

在总结成绩的同时，我们也清醒地认识到，我市经济社会发展还存在不少困难和问题，主要表现在：调整经济结构、推动产业优化升级、强化节能减排，还需要付出不懈努力；加快城乡一体化发展、改善农村牧区生产生活条件、缩小城乡差距，还需要下更大的力气；加强环境综合整治、全面推进生态建设、增强可持续发展能力，还需要谋求新的突破；深化改革开放、解决科技创新能力和人才资源相对不足的问题，还需要采取更加有效的举措；提高城市现代化水平、增强承载功能和辐射带动能力、确立区域性中心城市地位，还需要继续加快建设步伐；增加城乡居民收入、提高基本公共服务水平、着力保障和改善民生，还需要做大量扎实的工作；政府职能转变还需要进一步加快，发展环境还有待进一步优化。对于这些问题，我们一定要增强忧患意识和责任意识，采取有力措施，认真加以解决。

二、“十二五”时期经济社会发展的主要任务

“十二五”时期是我市加快推进富民强市进程、全面建设小康社会的关键时期，是深化改革开放、加快转变经济发展方式的攻坚时期。根据党的十七届五中全会、自治区党委八届十三次全委会议和市委《关于制定国民经济和社会发展第十二个五年规划的建议》精神，“十二五”时期我市经济社会发展的指导思想是：高举中国特色社会主义伟大旗帜，以邓小平理论和“三个代表”重要思想为指导，深入贯彻落实科学发展观，顺应各族人民过上更好生活的新期待，大力实施“科学发展、全面转型、富民强市”战略，以科学发展为主题，以加快转变经济发展方式为主线，以富民强市为目标，深化改革开放，保障和改善民生，加快推进新型工业化、城镇化、农牧业现代化，继续保持地区经济强劲发展态势，促进社会稳定和谐，推动经济、社会、生态全面转型，为提前实现全面建设小康社会目标奠定坚实的基础。

今后五年经济社会发展的主要目标是努力实现“三个提升”。一是提升地区综合经济实力。经济结构战略性调整取得实质性进展，经济增长的质量和效益进一步提升，确保增长速度高于全区平均水平，城市综合经济实力和综合竞争力在全国地级以上城市中的位次较快前移。二是提升城市品位和素质。城市规划和设计不断完善，空间布局更加优化，管理更加科学，城乡基础设施和综合服务功能更加完善，区域性中心城市的辐射带动作用明显增强。三是提升城乡居民物质文化生活水平。城乡居民收入增长和经济发展同步、劳动报酬增长和劳动生产率提高同步，覆盖城乡的社会保障体系和公共服务体系更趋完善，事关民生的紧迫性问题基本得到解决，民主法制建设继续加强，社会管理方式不断创新，社会和谐程度进一步提高。

具体预期指标是：地区生产总值年均增长15%左右，地方财政总收入年均增长16%，固定资产投资年均增长18%以上，社会消费品零售总额年均增长19%以上，进出口总额年均增长15%，城镇居民人均可支配收入和农牧民人均纯收入年均增长14%左右，城镇登记失业率年均控制在3.9%以内。各项节能减排指标达到自治区要求。

实现上述任务目标，必须加快推进“五个转变”。一是产业定位由老工业基地向现代产业基地转变。坚持走新型工业化道路，着力推进传统产业优化升级，积极培育发展战略性新兴产业，优化三次产业结构，构建现代产业体系。二是城市功能由功能相对单一的工业城市向区域性中心城市转变。在做大做强工业、提升产业集聚辐射能力的基础上，加快推进城市基础设施和环境建设，强化城市服务功能和综合承载能力，打造区域性中心城市。三是动力机制由主要依靠物质资源投入拉动向科技创新驱动转变。在继续优化投资结构、扩大投资规模的同时，推动经济增长更多依靠科技进步、劳动者素质提高和管理创新，加强人才高地和国家创新型城市建设。四是发展取向由更多地注重经济增长向更加关注改善民生转变。把保障和改善民生作为加快转变经济发展方式的根本出发点和落脚点，以促进就业、完善社会保障和基本公共服务体系为重点，不断提高城乡居民物质文化生活质量，让人民群众更多地享有改革发展成果。五是增长方式由粗放型增长向集约型增长转变。推动绿色发展、低碳发展、循环发展，加快转变高耗能、高排放、高污染的粗放型增长方式，努力形成节约资源、保护环境的产业结构、增长方式和消费模式，走可持续发展之路。

为此，我们要认真做好以下重点工作。

（一）大力发展非资源型产业，加快产业结构转型升级。着眼于把我市建设成为国家西部地区重要的冶金（有色）产业基地和北方地区重要的特色装备制造业基地，在增量中调整结构、在转型中优化升级，加快改造提升传统优势产业。重点发展重型汽车、矿用车、铁路车辆、工程机械等先进装备制造业，积极培育新能源、新材料、新型煤化工等战略性新兴产业。突出抓好现代物流、交通运输、金融等生产性服务业，积极发展商贸餐饮、文化旅游等生活性服务业，努力提高服务业的比重和水平。深入实施“收缩、转移、集中”、“南菜北薯、乳肉并举”和“品牌带动”战略，加大农牧业投入，强化科技、装备支撑，提高农牧业规模化、设施化、产业化、标准化水平，努力发展现代高效农牧业。

（二）推进城市现代化建设，着力打造生态宜居环境。坚持高起点规划，进一步完善城市总体规划，优化城市布局，积极构建“一市双城、带状组团、绿色隔离”的城市新形态。坚持高标准建设，统筹安排城市建设各类资源，做好城市设计，加大城市标志性工程建设力度，加快城市出入口景观改造，加强城市园林绿化和保护，努力打造精品建筑、精品绿地、精品道路和精品节点，提升城市品位。加快综合交通运输体系和城市重大基础设施建设，基本形成适应经济社会发展需要的现代基础设施体系。与城市防洪减灾、环境治理、水资源综合利用、生态建设相结合，建设城市水系景观，彰显城市灵气。进一步规范城市空间开发秩序，积极推进城市新区建设，实施东河老城区等重点区域改造和开发。主动融入以呼包鄂为核心的西部经济区发展，认真落实自治区沿黄河、沿交通干线经济带重点产业发展规划，加快与周边地区在重大基础设施等方面的衔接，建设区域性中心城市。

（三）统筹城乡协调发展，加快推进城乡一体化。按照政策引导、规划先行、重点突破、梯度推进的要求，统筹城乡发展，构建与经济社会发展相适应的新型城乡格局。制定完善土地管理、空间布局、产业发展、基础设施、公共服务、社会保障和社会管理“七个一体化”的专项规划，建立健全户籍管理、土地流转、公共财政等政策体系。大力发展县域经济，鼓励和推动旗县区因地制宜发展各具特色的优势产业，引导城区部分产业、重点项目向外围旗县区布局，培育形成县域经济发展的重要支柱。加强旗县区基础设施和产业园区建设，加速推进城镇化进程。建立以工促农、以城带乡长效机制，推动城镇基础设施向农村牧区延伸，促进城市公共服务向农村牧区覆盖，加快农牧民向城镇转移，稳步推进社会主义新农村新牧区建设。

（四）改善生态环境，努力建设生态文明。加大空气污染综合整治力度，力争空气质量二级以上良好天数占全年比例达到90%。加强水污染防治，实施污水零排放工程，确保在黄河上游来水达标的前提下包头段水质达到功能水体要求。加快水资源综合利用，积极推进“健康水”工程。大力推进生态建设和保护，继续实施国家重点生态建设项目。巩固山北草原围封禁牧成果，完善草原生态补偿机制，努力建设北部绿色生态屏障。加大节能减排力度，抓好重点行业、重点领域、重点企业的节能减排工作，确保完成自治区下达的控制性指标。

（五）加强以改善民生为重点的社会建设，不断提升人民群众的幸福感和满意度。加快分配制度改革，进一步拓宽增收渠道，千方百计增加城乡居民收入，不断增加低收入者收入，扩大中等收入群体。加快推进覆盖城乡的社会保障体系建设，在扩大覆盖范围、提高保障水平和统筹层次等方面迈出更大步伐。优先发展教育事业，进一步加大教育投入，深化教育教学改革，均衡办学条件，推动各级各类教育协调发展。深化医疗卫生体制改革，健全覆盖城乡的基本医疗卫生服务体系，提高人民群众的健康水平。全面实施保障性安居工程，努力实现住有所居、应保尽保。巩固扩大全国文明城市创建成果，加快发展文化事业和文化产业，努力把文化产业打造成为国民经济新的增长点。全面贯彻落实党的民族宗教政策，进一步巩固发展平等、团结、互助、和谐的民族关系。

（六）深入推进改革创新，构建全方位开放格局。进一步深化国有企业改革，深入推进行政管理体制改革，加快资源性产品价格和资源配置方式改革，深化农村牧区综合改革和统筹城乡综合配套改革。大力发展非公有制经济，提高非公有制经济在全市经济中的比重。着力推进原始创新、集成创新和引进消化吸收再创新，实施重大产业技术开发专项工程，加大对非资源型产业研发项目的扶持力度，强化企业创新主体地位，加快创新平台建设。认真实施自治区“草原英才”工程，培养和吸引各领域人才在包头创业。坚持扩大开放与区域协调发展相结合，“引资”与“引智”相结合，在引进战略投资、对接基础设施、承接产业转移、引进技术和管理等方面实现更大突破。进一步开拓国际市场，提升我市对外开放水平。全面履行政府职能，着力优化经济社会发展环境。

三、2011年工作安排

2011年是“十二五”开局之年，我们将迎来中国共产党成立90周年。做好政府各项工作，具有十分重要的意义。今年全市经济社会发展的主要预期目标是：地区生产总值增长16%左右，地方财政总收入增长17%，固定资产投资增长20%左右，社会消费品零售总额增长19%以上，城乡居民收入分别增长13%左右，城镇登记失业率控制在3.9%以内。完成自治区下达的节能减排指标。实现上述目标，要重点做好以下工作：

（一）调整优化工业经济结构，增强工业经济实力和竞争力。

全市规模以上工业增加值增长19%左右。实施投资5000万元以上工业重点项目323个，年内力争完成投资1000亿元以上，增长20%左右。

改造提升传统产业。实施投资5000万元以上传统产业改造升级工业重点项目158个，总投资549亿元，当年计划投资286亿元。钢铁产业方面，实施包钢高速重轨旧线改造、360万吨球团、1—4号焦炉节能环保和烧结系统搬迁改造等45个项目。有色产业方面，实施龙马铝业铝型材深加工、东方希望固阳10万吨镁合金及600万吨固体废弃物综合利用等24个项目。装备制造产业方面，实施北重集团6万吨精锻及配套生产、5000架液压支架煤机生产线二期改造，北方股份1000台非公路矿用车、北方奔驰5万台重卡总装生产线等89个项目。

发展战略性新兴产业。实施新兴产业项目90个，总投资830亿元，当年计划投资276亿元。制造业方面，实施众翔通用航空200架轻型飞机制造等21个项目，加快引进吉利汽车项目，积极发展电动汽车。大力开发稀土新材料在新能源、电子信息、冶金等领域的应用，实施包钢稀土3万吨高性能磁性材料等28个项目。加强稀土资源保护，加快交易平台和储备制度建设。新能源方面，实施华电巴音200兆瓦风电、山晟新能源多晶硅、单晶硅二期及400兆瓦太阳能组件生产线等19个项目，推进福日能（包头）高科八寸晶圆芯片等9个项目。新型化工产业方面，重点推进泛海集团360万吨煤制甲醇转120万吨烯烃等13个项目。

加快园区建设。按照沿黄河沿交通干线进行工业布局的要求，进一步明确园区发展定位，加强规划管理，强化产业分工协作，实现园区优势互补和错位发展，将我市各工业园区建设成为“一园一主业、主业特色化”的新型产业基地。建立工业园区投融资平台，多渠道筹措资金，加快园区供水、供电、供气等基础设施建设。完善园区在节能减排、投资强度和效益、管理服务等方面的评估考核。今年要引进实施243个项目，完成工业总产值1650亿元，增长20%。加强稀土高新区建设，按照科技含量高、产业关联度高、投入强度高、产出密度高的“四高”标准，加快打造以稀土新材料及稀土应用器件为主的高新技术产业集群，建设体制机制创新、节能环保和高效管理的示范区。

增强自主创新能力。加大科技投入，今年全社会研究与开发经费支出占全市GDP的比例达到1%，规模以上工业企业研究与开发经费占产品销售收入的1.2%以上。支持企业与科研院所和高校开展产学研合作，鼓励企业创造知识产权和制定技术标准。围绕优势特色产业和战略性新兴产业，实施一批重大科技项目，解决产业发展的技术瓶颈问题。加强国家重点实验室和实验基地建设，推进中科院、清华大学在包头设立分支机构，积极培养和引进高层次创新型人才和高技能型人才，推动科技创新和科技进步。大力实施品牌战略，鼓励企业争创各类名牌产品和中国驰名商标。全年组织实施20项重点科技项目，新增国家级高新技术企业10家以上，创建科技创新型试点企业20家，组织认定10个重点自主创新产品和10项节能减排示范技术，全市高新技术企业产值完成550亿元，同比增长20%。

支持和鼓励企业发展。加快培育一批主业突出、核心竞争力强、行业领先的大企业、大集团，推动东方希望铝业销售收入超过百亿元。大力推进自治区“促进中小企业政策落实年”各项工作，实施中小企业带动就业工程，引导中小企业围绕优势特色产业抓好产业延伸、围绕骨干企业抓好协作配套，推动中小企业转型升级，促进中小企业集群化发展。全力实施中小企业成长工程，加快中小企业创业园建设，进一步完善服务体系，着力解决融资难等问题，为中小企业发展创造更多有利条件。

（二）不断拓展服务业发展领域，全面提升服务业发展质量和水平。全市第三产业增加值增长17%，实施投资5000万元以上服务业重点项目387个，年内完成投资734亿元，增长20%左右。

突出抓好生产性服务业。按照“服务带动、物流先行”的思路，以城市统一配送、农村商贸物流、生产资料物流为重点，全面推进全国流通领域现代物流示范城市和区域性物流节点城市建设。积极构建“物流基地—物流园区—物流中心”网络，大力发展钢铁石化、装备机电、煤炭3个物流基地，稀土、有色金属、口岸等6个物流园区以及建材、粮油等9个物流中心，加快新奥蒙华、润恒、力德国际汽车城等项目建设，使物流业成为我市的主导产业之一。大力引进兴业银行、光大银行、民生银行以及外资银行，力争年内1—2家银行落地。支持保险、证券、典当、信用担保、小额贷款等其它金融业发展。加快会展中心建设，积极承办中国西部国际专利技术暨产品博览会等大型会展活动，推动会展经济发展。大力发展总部经济，推进律师、会计、咨询、评估、广告策划等中介服务业发展。

积极发展生活性服务业。重点培育批发、零售和住宿餐饮企业各50户，推动其营业额突破500亿元。积极引进奥特莱斯、巴黎春天等国内外知名品牌，加快建设凯旋中央公园、东正财富广场，确保金荣财富中心、东源国际年内投入使用。大力实施社区商业“双进工程”，引导企业运用连锁经营方式到社区设立超市、便利店、餐饮店、洗衣店等各类便民、利民网点。继续实施万村千乡市场工程、双百市场工程、家电下乡工程和以旧换新工程，积极推进“农超对接”工作。抓好昆区团结市场、青山互助道市场等8个集贸市场的升级改造，提升便民服务水平。加大对旅游业的投入，推动小白河湿地、希拉穆仁、五当召、秦长城等景区开发建设，加快开发麻池古城、满都拉口岸、乔家金街等旅游资源，提升包钢、北重等全国工业旅游示范点的影响力。举办中国·包头秦长城热气球节、中国·包头黄河湿地风情节等节庆活动，打造“草原休闲之都”。

（三）统筹城乡协调发展，加快推进农牧业现代化。第一产业增加值增长7%，实施投资5000万元以上重点农牧业和产业化项目17个，年内完成投资17.8亿元。

加快城乡一体化进程。全面实施城乡一体化发展规划，不断提高城乡统筹发展水平。完成城乡一体化建设规划的报批以及昆区北部区、110国道两侧等地区城市设计和新城村、昌福南村等8个“城中村”规划编制工作。完成乡镇总体规划和70%以上的行政村规划的编制工作。组织开展固阳县、达茂旗县域村镇体系规划编制工作。继续推进“城中村”、城乡结合部改造，巩固26个“城中村”改造成果，完成新城村等10个“城中村”改造建设任务。加快土右旗中等城市建设和固阳县、达茂旗、石拐区、白云矿区城镇建设。充分利用国家对农村牧区危房改造方面的资金补贴和优惠政策，抓好农村牧区村容村貌试点改造工作，全面推进村庄整治和环境治理。加快包头至固阳输供水管线工程建设，力争年底竣工。

大力发展现代农牧业。加强以水利为重点的农牧业基础设施建设，加快滴灌、喷灌等节水设施建设。抓好“菜篮子工程”，蔬菜保护地面积要新增3万亩，达到8.5万亩，新建大型交易市场1个、产地批发市场2个，提高蔬菜自给特别是冬春季供给能力。做大做强马铃薯产业，健全完善良种繁育、质量检测体系，积极引进扶持种薯生产、加工企业，延伸产业链条。设施马铃薯面积新增17万亩，达到70万亩，其中滴灌面积新增14万亩，达到30万亩。依托小肥羊、小尾羊等“群羊”产业，扩大养殖规模，加快品种改良，提升产业档次，力争羊存栏和出栏分别达到245万只和400万只。推动奶牛业持续健康发展，提高奶牛规模化养殖水平。完善农业技术服务体系，加强农产品标准、信息、质量检测监管和动植物疫病防控体系建设，保障农畜产品质量安全。

有效提高农牧民收入水平。严格落实国家、自治区和我市出台的粮食、良种、农机购置、农资综合补贴和围封禁牧等惠农惠牧政策，增加农牧民政策性收入。落实和健全草原生态补偿机制，积极争取国家和自治区资金及项目支持，实现草原生态保护建设和牧民稳定增收双赢。加强农村牧区富余劳动力转移就业的职业技能培训，大力发展劳务经济，提高劳务输出的组织化程度，增加农牧民工资性收入。切实加大扶贫开发投入，重点实施整村推进、产业化扶贫项目，增加贫困地区群众收入。继续做好集中社会力量重点帮扶固阳县工作，转变帮扶形式，整合各类资金，突出产业帮扶。千方百计拓宽农牧民增收渠道，分解落实地区、部门责任，确保农牧民收入目标如期实现。

（四）深化改革开放，增强经济社会发展活力。积极稳妥地推进各项改革，力争在重点领域和关键环节取得新进展，大力发展外向型经济，打造自治区向北开放的前沿。

坚持推进各项改革。完善企业国有资产监管体系，切实履行政府出资人职责，保证国有资产保值增值。积极深化事业单位改革，创新管理体制，健全完善激励竞争机制，提高公共服务水平。严格预算管理，拓宽筹资渠道，合理安排财政支出，保证法定支出和重点支出增长。

加大招商引资力度。进一步创新招商方式，充分发挥企业主体作用，面向长三角、珠三角、京津冀、港澳台、陕甘宁等地区，积极开展专题招商、重点招商和小团组招商，认真落实好自治区政府组织的赴香港招商任务，有针对性地与世界和国内500强等大企业集团对接洽谈，在寻求战略投资者上实现新突破。推动大企业之间、驻包企业与地方之间广泛开展合作，促进融合发展。加强诚信体系建设，提高服务水平，营造宜商发展环境。继续实行领导分包重点项目制度，完善重大签约项目分级跟踪落实责任制，切实提高签约项目的合同履约率、资金到位率和项目开工率。今年，引进国内（区外）资金要达到360亿元。

发展壮大非公有制经济。认真贯彻落实国务院关于鼓励和引导民间投资健康发展的若干意见，完善促进非公有制经济发展的配套政策，健全创业辅导、融资担保、技术支持等服务体系，创造公平竞争、平等准入的市场环境。帮助支持有条件的民营企业上市融资、发行债券。发挥非公有制经济扩大就业、增加居民收入、拉动消费的作用。今年，非公有制经济占到地区生产总值的比重要达到51%。

提高对外开放水平。充分利用国内外两个市场、两种资源，转变对外贸易增长方式，提高利用外资的质量和水平。实施“走出去”和“向北开放”战略，推动与俄罗斯和蒙古国的经贸合作。鼓励支持有比较优势的企业开拓国际市场，拓展对外经济合作领域。加快满都拉口岸、集装箱中转站建设和包头机场扩建，打造全方位对外开放大通道和战略节点。今年，进出口总额要达到22.4亿美元，增长15%；实际利用外资要达到12.4亿美元，增长15%。

（五）加强城乡建设管理，建设生态宜居城市。全面抓好城市基础设施建设和环境建设，着力提升服务功能和辐射带动能力，为“十二五”末建成区域性中心城市奠定基础。

高起点做好城市规划设计。按照统筹规划、合理布局、重点突出、适度超前的要求，进一步提高城市规划区内控规覆盖率，年内实现规划建设用地范围322平方公里控规全覆盖。做好钢铁大街沿线商业区、民族东路两侧、210国道南段两侧、北梁地区、南海地区、九原北部区、小白河地区等重点区域规划。认真做好钢铁大街沿线、阿尔丁大街等重点区域、重要街区、重要节点的形象设计，集中力量打造精品城区，提升城市整体形象。

高强度推进城市建设。全年实施城建项目158个，总投资751.9亿元，年内计划投资309.2亿元。实施旧小区、小街巷以及小区供热二次管网和污水管网改造工程，着力解决热源不足、热网保障的问题。加快城市供水管网改造，保障城市供水安全。推进台地公园、奥林匹克公园建设，全年新植大规格树木35万株。加大城区“五横八纵”路网建设力度，实施一批道路新建改造工程，构建市区半小时、市域1小时交通圈。推动110国道协力气至西北门段、省道211线固阳至百灵庙、包头至石拐一级公路、省道104线百灵庙至白云鄂博等公路改建项目建设，尽早实现市区到旗县区的一级公路通达。实施南绕城、包钢厂前路、包钢西路、白云路连接包固一级公路道路建设。做好机场迁建、城市轨道交通项目的立项、报批等前期工作。新购置50台节能环保型公交车，方便群众出行。加大新都市中心区的征拆和建设力度，推进滨河新区、青山北部区、昆区南部区和北部区、石拐南部区建设，加快东河北梁地区改造。

精细化管理城市。加强城市管理综合执法，严厉打击未批先建行为，巩固整治违法建设成果。以户外广告、店招牌匾、夜景亮化为重点，加快市容环境整治，美化街容街貌。加强市政、园林设施监管，逐步建立公众参与的城市管理机制。强化城乡公共卫生管理，打好创建国家卫生城市攻坚战，为创建国家环保模范城奠定基础。

加强环境保护和生态建设。抓好南海子、小白河、白银湖等重点沿黄湿地的保护利用和生态恢复，加快包头黄河国家级湿地公园申报进度。强化禁牧督查和草原治理，加快推进舍饲圈养。继续组织实施天然林保护、京津风沙源治理、退耕还林等国家林业重点工程以及城郊结合部绿化、村屯绿化工程，进一步改善生态环境。加大对重点区域、重点企业，以及城区燃煤锅炉、建筑工地扬尘等空气污染治理力度，大力推广清洁能源，新建餐厨垃圾和建筑垃圾处理厂各一座，扩建东河垃圾卫生填埋场。今年空气质量二级以上良好天数达到320天。加快建设包铝、希铝、一机、北重、华资实业等工业废水综合治理工程。新建改扩建一批城区污水处理厂和污泥处理厂，年内生活污水日处理能力达到43万吨，处理率达到85%。强化东河、二道沙河、四道沙河、昆河环境综合治理，年内完成10—20公里河道整治任务。完成小白河应急防凌蓄洪区建设。划定集中饮用水源地保护区范围，清理保护区内违章建筑和排污口，确保饮用水源安全。启动“三片两线”规划整治工作，加快淘汰落后产能，该改造的企业彻底改造，该搬迁的企业必须搬迁，该取缔的企业坚决取缔。推进资源节约和综合利用，实施冶金渣、粉煤灰、选矿尾矿等固体废弃物处置工程，处置利用率达到90%以上。继续清理整顿城市周边采石场、采砂场、料场和建筑工地，加强对闲置土地的清理处置。积极开展节水、节材、节能活动，完成100万平方米既有居住建筑节能改造。

（六）努力保障和改善民生，全面推进和谐包头建设。加强以改善民生为重点的社会建设，维护好人民群众的切身利益，不断满足人民群众对社会公共服务的需求。

立足民需，协调发展各项社会事业。一是优先发展教育。全面实施素质教育，提高义务教育教学质量。继续落实好经济困难家庭学生享受国家助学金和免学费政策。提高高中教育普及水平，为三年内实现高中阶段免费教育奠定基础。加强教师队伍建设，加大教师培训力度。加强学前教育，新建10座公立幼儿园。大力发展职业教育，优化专业课程体系，年内职业教育基地一期工程投入使用。全面完成中小学校舍安全工程建设任务，推进办学条件标准化、均衡化。重视民族教育，整体搬迁蒙古族中学，年底建成并交付使用，启动回民中学搬迁工程，年内主体工程完工。做好铁一中、铁二中的搬迁工作。二是加强公共卫生和医疗服务体系建设。加快医疗卫生体制改革，调整和优化医疗卫生资源配置，提高社区、村镇卫生服务能力。继续开展扶贫和康复诊疗活动，进一步扩大“健康包头行动”的参与范围和服务内容，不断增强医疗惠民成效。强化医疗机构医疗质量、安全服务和绩效评价体系建设，努力构建和谐医患关系。三是加快文化大市建设。制定出台《包头市“十二五”文化产业发展规划》。进一步完善基层文化馆、图书馆、特色博物馆、综合文化站等文化基础设施建设，重点加快社区、村文化室和“草原书屋”建设。新图书馆、少年宫、科技馆尽快投入使用。积极开展群众文化活动，促进城乡群众文化繁荣发展。加强文化遗产保护工作。积极推进内蒙古文化传媒创意园区、敕勒川文化产业园区、燕家梁文化产业园区、包头市秦长城文化产业园区、天龙文化产业园区等重点文化建设项目，提高我市文化产业的影响力和竞争力。四是大力发展体育事业。加快体育场、乒羽馆、游泳馆、网球中心等场馆建设，办好第十一届全国中学生运动会。积极备战全国第七届城市运动会和自治区第十三届全运会，不断提升竞技体育水平。发展群众体育，大力开展丰富多彩的全民健身活动。五是全面推进各项事业发展。完善“村村通”广播电视工程，组织好农村牧区广播电视数字村建设。继续实施农村牧区电影放映和送节目下基层工程。加强人口和计划生育工作，继续稳定低生育水平，提高出生人口素质。抓好全民国防教育，加强国防后备力量建设，巩固双拥模范城创建成果。支持工会、共青团、妇联、残联、红十字会等人民团体工作。抓好审计、统计、人防、地震、档案、气象、外事侨务、史志等各项工作。

关注民生，发展成果让人民群众共享。一是完善就业服务体系。全面落实各项优惠政策，以建设创业型城市为抓手，推动创业带动就业，促进下岗失业人员再就业，重点做好高校毕业生和城镇就业困难人员的就业援助工作，确保全年实现城镇新增就业4万人。二是完善社会保障体系。继续扩大城镇职工基本养老、医疗、失业、工伤和生育保险覆盖面，规范养老保险、医疗保险市级统筹。完善城镇居民基本医疗保险制度，提高新型农村牧区合作医疗参合率和最高支付限额。全面推行新型农村牧区社会养老保险制度，逐步建立“以社会保险为基础，基本养老、基本医疗为重点，商业保险为补充”覆盖城乡的社会保险体系。三是完善社会救助体系。切实推进城乡社会救助一体化进程，确保全市城乡低保对象救助水平稳步提高。加快完善城乡医疗救助制度，加大对困难群体的法律援助力度，做到应援尽援。加强敬老院建设，倡导以居家养老为基础，以社区服务为依托，以养老服务机构为补充的多样化养老服务体系。切实做好防灾救灾工作，保障受灾群众的基本生活不受影响。四是完善住房保障体系。建设经济适用住房26886套、廉租住房7081套、公共租赁房8619套，改造棚户区3.87万户，着力解决好中低收入家庭和困难群众的住房问题，促进全市房地产市场健康发展。五是解决好人民群众关注的热点难点问题。把更多的人力、物力、财力投入到保障和改善民生上来，围绕就业、社会保障、公共服务、改善环境等方面，继续为人民群众兴办20件32项好事实事，让人民群众在改革发展中得到更多实惠。加强物价管理，落实好中央和自治区稳定物价各项政策，逐步建立社会救助和保障标准与物价上涨挂钩的联动机制，确保低收入群众生活水平不因物价上涨而降低。

保障民安，促进社会和谐稳定。一是高度重视信访工作。健全接访中心运行机制，完善社会矛盾纠纷排查化解工作体系，切实落实好自治区出台的涉及政策层面信访问题的解决方案，维护人民群众的切身利益。二是加强安全生产监管。建立健全安全生产综合协调、激励约束、联合执法、事故查处与责任追究等工作机制，加强矿山、危险物品、建筑施工、消防、交通、特种设备等重点行业和领域的安全监管，有效遏制重特大安全事故。三是健全应急管理体制。加强城市防灾体系建设，建立健全社会预警体系和应急救援、动员机制，提高处置突发事件能力。四是加强食品药品安全监管。健全食品药品安全监管工作长效机制，继续深入开展食品药品安全专项整治行动，严格进行质量监督，切实保障人民群众饮食用药安全。五是强化社会治安综合治理。健全社会治安防控体系，推进和谐社区、和谐乡村建设，依法防范和打击违法犯罪活动，保障人民生命财产安全。六是切实加强民族宗教工作。全面落实党的民族宗教政策，大力开展民族团结进步宣传教育和创建活动，依法加强宗教事务管理，巩固和发展安定团结的大好局面。

（七）加强政府自身建设，更好地服务于经济社会发展。按照为民、务实、高效、廉洁的要求，强化依法行政，加快转变政府职能，提高社会管理科学化水平，努力建设法治政府、服务政府、责任政府和效能政府。

深入推进民主法制建设。认真执行人大及其常委会的决议和决定，及时报告工作，自觉接受监督。积极支持人民政协履行政治协商、民主监督、参政议政的职能，主动听取各民主党派、工商联、无党派人士及各人民团体的意见和建议。认真办理人大代表议案、建议和政协提案，不断提高办结率和满意度。深化行政审批制度改革，进一步减少行政审批事项和行政事业性收费。加快市政府政务服务中心建设，力争年底前竣工投入使用。健全社会公示、社会听证等制度，强化新闻舆论和社会公众监督，让人民群众更广泛地参与公共事务管理。扩大政务公开，加快电子政务建设，增强政府工作的透明度，提高政府公信力。

切实加强政风建设。认真落实党风廉政建设责任制，严格执行领导干部廉洁自律的各项规定，健全完善惩治和预防腐败体系。充分发挥监察、审计部门作用，注重结果运用，坚持用制度管权、管人、管事，强化对权力运行的监督。树立过紧日子的思想，厉行节约，反对浪费，把有限的资金更多地用于促进发展和改善民生。加强公务员教育培训和监督管理，严肃查处违反行政纪律和侵害人民群众利益的案件，坚决纠正损害群众利益的不正之风，树立良好的政府形象。

狠抓工作落实。牢固树立公仆意识和责任意识，坚持深入基层、深入实际，问政于民，问计于民，问需于民，努力为基层和群众解决实际问题。始终保持锐意进取、奋发有为的精神状态，深入落实好中央、自治区和市委的决策部署，讲实话、办实事、求实效，全身心地投入到“科学发展、全面转型、富民强市”的实践中，高质量、高效率、高水平地做好政府的各项工作。

各位代表！包头正在崛起，前景催人奋进。我们要紧密团结在以胡锦涛同志为总书记的党中央周围，在自治区党委、政府和包头市委的领导下，倍加珍惜不可多得的重要机遇，倍加珍惜来之不易的发展势头，开拓创新，真抓实干，为实现全面建设小康社会的宏伟目标而努力奋斗！
